# Gene array identification of *Ipf1/Pdx1*^-/- ^regulated genes in pancreatic progenitor cells

**DOI:** 10.1186/1471-213X-7-129

**Published:** 2007-11-23

**Authors:** Per Svensson, Cecilia Williams, Joakim Lundeberg, Patrik Rydén, Ingela Bergqvist, Helena Edlund

**Affiliations:** 1 Umeå Center for Molecular Medicine, Umeå University, SE-901 87 Umeå, Sweden; 2 School of Biotechnology, KTH, Royal Institute of Technology, AlbaNova University Center, SE-10691 Stockholm, Sweden; 3 Department of Biosciences at Novum, Karolinska Institutet, 14157 Huddinge, Sweden; 4 Department of Mathematics and Mathematical Statistics, Umeå University, SE-901 87 Umeå, Sweden Department of Clinical Bacteriology, Universityhospital, SE-90185 Umeå, Sweden; 5 Betagenon AB, Box 7966, SE-907 19 Umeå, Sweden

## Abstract

**Background:**

The homeodomain transcription factor IPF1/PDX1 exerts a dual role in the pancreas; *Ipf1/Pdx1 *global null mutants fail to develop a pancreas whereas conditional inactivation of *Ipf1/Pdx1 *in β-cells leads to impaired β-cell function and diabetes. Although several putative target genes have been linked to the β-cell function of *Ipf1/Pdx1*, relatively little is known with respect to genes regulated by IPF1/PDX1 in early pancreatic progenitor cells.

**Results:**

Microarray analyses identified a total of 111 genes that were differentially expressed in e10.5 pancreatic buds of *Ipf1/Pdx1*^-/- ^embryos. The expression of one of these, *Spondin 1*, which encodes an extracellular matrix protein, has not previously been described in the pancreas. Quantitative real-time RT-PCR analyses and immunohistochemical analyses also revealed that the expression of *FgfR2IIIb*, that encodes the receptor for FGF10, was down-regulated in *Ipf1/Pdx1*^-/- ^pancreatic progenitor cells.

**Conclusion:**

This microarray analysis has identified a number of candidate genes that are differentially expressed in *Ipf1/Pdx1*^-/- ^pancreatic buds. Several of the differentially expressed genes were known to be important for pancreatic progenitor cell proliferation and differentiation whereas others have not previously been associated with pancreatic development.

## Background

The pancreas is an endodermally derived organ that forms from a ventral and a dorsal evagination of the foregut epithelium. These two evaginations, the dorsal and ventral pancreatic buds, subsequently grow, branch and differentiate into distinct pancreatic cell types [1]. The homeodomain transcription factor Insulin Promoter Factor 1/Pancreatic and Duodenal homeobox 1 (IPF1/PDX1) is one of the earliest markers of the developing pancreas. IPF1/PDX1 is expressed already at ~10 somites stage at the regions of the dorsal and ventral gut endoderm from which the pancreatic buds evaginate [2]. IPF1/PDX1 expression remains high in pancreatic epithelial cells until ~e10.5 after which it is down-regulated [3] and remains low in proliferating pancreatic epithelial cells. Strong IPF1/PDX1 expression reappears in the differentiating β-cells as they emerge at ~e13 [3] and high level of IPF1/PDX1 expression is maintained in adult β-cells where IPF1/PDX1 controls the expression of several key β-cell genes, including the insulin gene, thereby ensuring normal β-cell function and glucose homeostasis [4,5].

Loss of *Ipf1/Pdx1 *gene function in mice and humans results in pancreatic agenesis demonstrating a key role for the *Ipf1/Pdx1 *gene in pancreatic development [6-8]*. Ipf1/Pdx1 *is, however, not required for the initiation of the pancreatic program and the initial stages of pancreas development, i.e. the formation of the pancreatic buds, still occurs in *Ipf1/Pdx1*^-/- ^mice [7,9]. Although the pancreatic program is initiated in *Ipf1/Pdx1 *deficient embryos, the subsequent growth of the embryonic pancreas is arrested, resulting in pancreas agenesis [6,7,9]. Recombination experiments between pancreatic epithelium and pancreatic mesenchyme have demonstrated that the pancreatic developmental defect observed in *Ipf1/Pdx1*^-/- ^embryos is confined to the epithelial cells [9]. Thus, pancreatic mesenchyme isolated from *Ipf1/Pdx1*^-/- ^e10.5 dorsal pancreatic buds could support the growth of wt e10.5 dorsal pancreatic epithelium whereas the reverse combination failed to grow [9]. These data provide evidence for a cell-autonomous role for *Ipf1/Pdx1 *in early pancreatic progenitor cells. To date, no direct or indirect *Ipf1/Pdx1 *downstream genes have, however, been identified that can explain the pancreatic phenotype observed in *Ipf1/Pdx1*^-/- ^embryos.

To identify *Ipf1/Pdx1 *target genes in early pancreatic progenitor cells and to begin to unravel the molecular mechanisms that lead to the attenuation of pancreatic growth in *Ipf1/Pdx1*^-/- ^mice we performed microarray analyses on cDNA prepared from *Ipf1/Pdx1*^-/- ^e10.5 buds and stage matched littermate wildtype controls. We have identified genes that are differentially expressed in *Ipf1/Pdx1*^-/- ^pancreatic buds and a subset of these was chosen for further expression analysis by quantitative real-time (qRT) RT-PCR, *in situ *hybridization and immunohistochemistry. In agreement with the pancreatic developmental defect observed in *Ipf1/Pdx1*^-/- ^embryos, several of the differentially expressed genes identified in this study encode factors linked to pancreatic progenitor cell proliferation and differentiation.

## Results

### Gene expression changes in *Ipf1/Pdx1*^-/- ^pancreatic buds

In order to identify candidate *Ipf1/Pdx1 *downstream genes in early pancreatic progenitor cells, dorsal pancreatic buds were isolated from e10.5 *Ipf1/Pdx1*^-/- ^and *Ipf1/Pdx1*^+/+ ^littermate embryos. cDNA was prepared from pancreatic buds derived from 4 independent *Ipf1/Pdx1*^-/- ^and 4 independent *Ipf1/Pdx1*^+/+ ^littermates respectively, labeled and hybridized to two different sets of microarrays. The first contain approximately 15,000 clones obtained through large-scale, in-house EST sequencing of three cDNA libraries originating from a neural tissue stem cell compartment (lateral ventricular wall), neurospheres (neural stem cells cultured in vitro), and a hematopoietic stem cell line expressing the Lhx2 gene [10]. The second cDNA array used in this study contains 20,600 clones derived from two different clone sets: a 15,000 mouse cDNA set from National Institute of Aging (NIH) and a 5,400 cDNA clone set obtained from Research Genetics. Genes that showed a change in expression that was two-fold or more and had sufficiently high test-statistics were considered to be differentially expressed (see Methods for details). The microarray analyses revealed a total number of 111 genes that were differently expressed. Of these 73 were down-regulated (Table 1) and 38 were up-regulated (Table 2) in e10.5 dorsal pancreatic buds of *Ipf1/Pdx1 *deficient mice as compared to that of stage matched wildtype littermates.

**Table 1:** Top ranked 73 down-regulated genes in *Ipf1/Pdx1*^-/- ^vs. *Ipf1/Pdx1 *^+/+ ^e10.5 dorsal pancreatic buds

**Genbank acc. No**.	**Gene Symbol**	**Gene Name**	**Fold-change**
**Extracellular matrix proteins**			
NM_009263	*Spp1*	secreted phosphoprotein 1	-9.8 U *
NM_033525	*Npnt*	Nephronectin	-6.1 **U**
NM_145584	*Spon1*	spondin 1, (f-spondin) extracellular matrix protein	-3.8 K **
			
**Transport**			
NM_172479	*Slc38a5*	solute carrier family 38, member 5	-8.8 **U**
NM_011401	*Slc2a3*	solute carrier family 2 (facilitated glucose transporter), member 3	-2.6 **K**
NM_009593	*Abcg1*	ATP-binding cassette, sub-family G (WHITE), member 1	-2.3 **U**
			
**Signaling**			
NM_018763	*Chst2*	carbohydrate sulfotransferase 2	-4.5 **U**
XM_618828	*Erbb3*	v-erb-b2 erythroblastic leukemia viral oncogene homolog 3 (avian)	-3.2 U *
NM_019946	*Mgst1*	microsomal glutathione S-transferase 1	-3.2 K *
NM_152815	*Lins2*	Lines homolog 2 (Drosophila)	-2.4 **U**
NM_025505	*Blzf1*	basic leucine zipper nuclear factor	-2.0 **K**
			
**Transcription factors**			
NM_013627	*Pax6*	paired box gene 6	-5.8 K *
NM_144955	*Nkx6-1*	Nkx transcription factor related, locus 1 (Drosophila)	-4.2 U ***
NM_008686	*Nfe2l1*	nuclear factor, erythroid derived 2,-like 1	-2.6 **U**
NM_010938	*Nrf1*	nuclear respiratory factor 1	-2.2 **U**
			
**Cell adhesion**			
NM_008532	*Tacstd1*	tumor-associated calcium signal transducer 1	-3.2 **U**
NM_009864	*Cdh1*	cadherin 1	-2.6 **U**
			
**Cell cycle**			
NM_013928	*Schip1*	schwannomin interacting protein 1	-3.1 **K+U**
NM_007631	*Ccnd1*	cyclin D1	-2.6 **U**
			
**DNA binding**			
NM_198169	*Gmeb2*	glucocorticoid modulatory element binding protein 2	-2.9 **U**
NM_175074	*Hmgn3*	high mobility group nucleosomal binding domain 3	-2.4 **K**
			
**Kinases/Phosphatases**			
NM_152804	*Plk2*	polo-like kinase 2 (Drosophila)	-2.9 **K**
NM_011950	*Mapk13*	mitogen activated protein kinase 13	-2.5 **U**
NM_008981	*Ptprg*	protein tyrosine phosphatase, receptor type, G	-2.4 **U**
			
**Apoptosis**			
NM_008410	*Itm2b*	integral membrane protein 2B	-2.3 **U**
			
**Other**			
NM_026433	*1810057C19Rik*	RIKEN cDNA 1810057C19 gene	-6.3 **U**
NM_153457	*Rtn1*	reticulon 1	-6.1 U *
NM_018737	*Ctps2*	cytidine 5'-triphosphate synthase 2	-5.9 **U**
NM_007694	*Chgb*	chromogranin B	-5.9 **K**
NM_026238	*Narfl*	nuclear prelamin A recognition factor-like	-4.9 **U**
NM_145562	*9130213B05Rik*	RIKEN cDNA 9130213B05 gene	-4.8 **U**
NM_011255	*Rbp4*	retinol binding protein 4, plasma	-4.2 **U**
NM_178747	*Gulo*	gulonolactone (L-) oxidase	-4.2 **U**
NM_025429	*Serpinb1a*	serine (or cysteine) proteinase inhibitor, clade B, member 1a	-4.2 **U**
AI451390		ESTs, transcribed locus	-4.0 **U**
NM_133977	*Trf*	Transferring	-4.0 **K**
NM_009412	*Tpd52*	tumor protein D52	-3.8 **U**
NM_016962	*Spast*	Spastin	-3.5 **U**
NM_015784	*Postn*	periostin, osteoblast specific factor	-3.3 **U**
NM_133697	*1110003E01Rik*	RIKEN cDNA 1110003E01 gene	-3.2 **U**
NM_145599	*Tmem34*	transmembrane protein 34	-3.1 **U**
NM_025633	*Metapl1*	methionine aminopeptidase-like 1	-3.0 **U**
XM_125538	*Sesn1*	Sestrin 1	-3.0 **K**
NM_013471	*Anxa4*	annexin A4	-3.0 **K**
NM_008188	*Thumpd3*	THUMP domain containing 3	-2.8 **U**
NM_026323	*Wfdc2*	WAP four-disulfide core domain 2	-2.7 **U**
NM_033073	*Krt2-7*	keratin complex 2, basic, gene 7	-2.7 **U**
BB529246		ESTs	-2.7 **K**
NM_178600	*Vkorc1*	vitamin K epoxide reductase complex, subunit 1	-2.6 **U**
NM_026524	*Mid1ip1*	Mid1 interacting protein 1 (gastrulation specific G12-like (zebrafish))	-2.6 **K+U**
NM_008792	*Pcsk2*	proprotein convertase subtilisin/kexin type 2	-2.6 **K**
NM_013778	*Akr1c13*	aldo-keto reductase family 1, member C13	-2.6 **K**
NM_011464	*Spint2*	serine protease inhibitor, Kunitz type 2	-2.5 **U**
NM_197996	*Tspan15*	tetraspanin 15	-2.5 **U**
NM_022316	*Smoc1*	SPARC related modular calcium binding 1	-2.5 **U**
NM_019972	*Sort1*	sortilin 1	-2.4 **U**
NM_176860	*2810457I06Rik*	RIKEN cDNA 2810457I06 gene	-2.4 **U**
XM_133714	*4921517J23Rik*	RIKEN cDNA 4921517J23 gene	-2.4 **K**
NM_146057	*Dap*	death-associated protein	-2.4 **K**
NM_013492	*Clu*	clusterin	-2.3 **U**
NM_198654	*4833432M17Rik*	RIKEN cDNA 4833432M17 gene	-2.3 **U**
NM_138651	*Cds2*	CDP-diacylglycerol synthase (phosphatidate cytidylyltransferase) 2	-2.2 **U**
NM_145991	*Hrpt2*	hyperparathyroidism 2 homolog (human)	-2.2 **U**
NM_010485	*Elavl1*	ELAV (embryonic lethal, abnormal vision, Drosophila)-like 1	-2.2 **U**
AI451897		ESTs, transcribed locus	-2.2 **U**
NM_029341	*Capsl*	calyphosine-like	-2.2 **K**
NR_001592	*H19*	H19 fetal liver mRNA	-2.1 **U**
XM_110660	*AI427122*	expressed sequence AI427122	-2.1 **U**
NM_025853	*1700022L09Rik*	RIKEN cDNA 1700022L09 gene	-2.1 **U**
NM_138606	*Pim2*	proviral integration site 2	-2.1 **K**
NM_031170	*Krt2-8*	Keratin complex 2, basic, gene 8	-2.0 **U**
NM_025975	*Tcte1l*	t-complex-associated-testis-expressed 1-like	-2.0 **K**
NM_007657	*Cd9*	CD9 antigen	-2.0 **K**

K = changes obtained from the 15 K microarray. U = changes obtained from the 21 K microarray. * = validated by qRT RT-PCR. ** = validated by qRT RT-PCR and *in situ *hybridization. *** = validated by qRT RT-PCR and immunohistochemistry.

**Table 2:** Top ranked 38 up-regulated genes in *Ipf1/Pdx1*^-/- ^vs. *Ipf1/Pdx1*^+/+ ^e10.5 dorsal pancreatic buds

**Genbank acc. No**	**Gene Symbol**	**Gene Name**	**Fold-change**
**Signaling**			
NM_009144	*Sfrp2*	secreted frizzled-related sequence protein 2	2.3 **U**
NM_175186	*Lin9*	lin-9 homolog (C. elegans)	3.6 **U**
NM_013834	*Sfrp1*	secreted frizzled-related sequence protein 1	3.7 **K**
			
**Cell cycle**			
NM_026410	*Cdca5*	cell division cycle associated 5	2.2 **U**
			
**Other**			
NM_011808	*Ets1*	E26 avian leukemia oncogene 1, 5' domain	2.0 **K**
NM_026421	*2310057D15Rik*	RIKEN cDNA 2310057D15 gene	2.0 **U**
NM_145397	*BC002059*	cDNA sequence BC002059	2.1 **U**
NM_007896	*Mapre1*	microtubule-associated protein, RP/EB family, member 1	2.1 **U**
NM_010942	*Nsg1*	neuron specific gene family member 1	2.1 **K**
NM_134022	*6330403K07Rik*	RIKEN cDNA 6330403K07 gene	2.1 **K**
BG075563	*Dio3as*	deiodinase, iodothyronine type III, antisense	2.2 **U**
NM_010923	*Nnat*	neuronatin	2.2 **K**
AI449753	*AI449753*	expressed sequence AI449753	2.2 **U**
NM_053197	*Sfxn3*	sideroflexin 3	2.2**U**
XM_620539	*Trim6*	tripartite motif protein 6	2.3 **U**
NM_177916	*C230004F18Rik*	RIKEN cDNA C230004F18 gene	2.4**U**
NM_173750	*2700007P21Rik*	RIKEN cDNA 2700007P21 gene	2.4 **U**
NM_024217	*Cklfsf3*	chemokine-like factor super family 3	2.4 **U**
NM_145629	*Pls3*	plastin 3 (T-isoform)	2.4 **U**
NM_007837	*Ddit3*	DNA-damage inducible transcript 3	2.4 **U**
NM_054082	*Mta3*	metastasis associated 3	2.5 **U**
NM_016797	*Stx7*	syntaxin 7	2.6 **U**
NM_008590	*Mest*	mesoderm specific transcript	2.6 **K**
NM_011058	*Pdgfra*	platelet derived growth factor receptor, alpha polypeptide	2.7 **K**
NM_012005	*Crsp2*	cofactor required for Sp1 transcriptional activation, subunit 2	2.7 **U**
AK037782	*D130019J16Rik*	RIKEN cDNA D130019J16 gene	2.7 **U**
NM_025783	*Vps24*	vacuolar protein sorting 24 (yeast)	2.9 **U**
NM_001004155	*9930012K11Rik*	RIKEN cDNA 9930012K11 gene	3.0 **U**
NM_146153	*Thrap3*	Thyroid hormone receptor associated protein 3	3.1 **U**
NM_175439	*Mars2*	methionine-tRNA synthetase 2 (mitochondrial)	3.2 **U**
NM_008865	*Csh2*	chorionic somatomammotropin hormone 2	3.2 **U**
NM_178631	*0710005M24Rik*	RIKEN cDNA 0710005M24 gene	3.3 **U**
NM_019721	*Mettl3*	methyltransferase-like 3	3.3 **U**
NM_030704	*Hspb8*	heat shock 27 kDa protein 8	4.0 **U**
NM_177648	*Tmem15*	transmembrane protein 15	4.1 **U**
NM_178608	*D6Ertd253e*	DNA segment, Chr 6, ERATO Doi 253, expressed	4.1 **U**
NM_024251	*2010301N04Rik*	RIKEN cDNA 2010301N04 gene	4.1 **U**
NM_010910	*Nefl*	neurofilament, light polypeptide	7.3 **U**

K = changes obtained from the 15 K microarray. U = changes obtained from the 21 K microarray.

### Altered expression of genes associated with cell proliferation and differentiation

Given the pancreatic growth arrest phenotype of *Ipf1/Pdx1*^-/- ^mice we selected a total of 10 differentially expressed genes identified by the microarray (9 down-regulated and 1 up-regulated) that encode factors linked to growth and differentiation for further expression analyses and validation by quantitative real-time RT-PCR. The selected genes include genes previously associated with pancreatic progenitor cell proliferation and differentiation, including *NK6 transcription factor related, locus 1 *(*Nkx6.1*), *v-erb-b2 erythroblastic leukemia viral oncogene homolog 3 *(*ErbB3*), *Secreted phosphoprotein 1 *(*Spp1) *and *Paired box gene 6 *(*Pax6)*, as well as genes encoding factors that previously have not been linked to pancreas development, such as *Spondin 1 *(*Spon1*), *Microsomal glutathione S-transferase 1 *(*Mgst1*), *Reticulon 1 *(*Rtn1*), *Basic leucine zipper nuclear factor 1 *(*Blzf1*), *Schwannomin interacting protein 1 *(*Schip1*), and *Secreted frizzled related protein1 *(*Sfrp1*).

**Figure 1:**
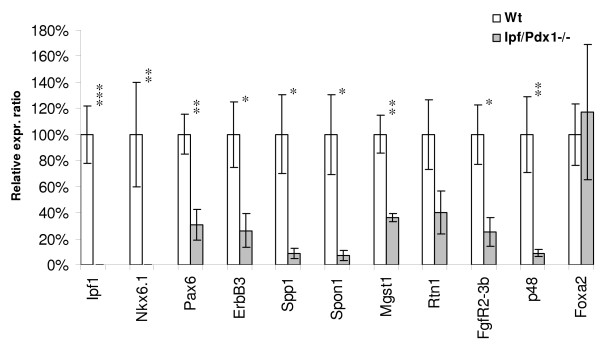
**Quantitative real-time RT-PCR validation of selected genes**. Expression analyses of the indicated genes using cDNA prepared from *Ipf1/Pdx1*^+/+ ^(n = 4–9) and *Ipf1/Pdx1*^-/- ^(n = 4–8) dorsal e10.5 pancreatic buds. Data represent mean values ± SEM. * p < 0.05; ** p < 0.01; *** p < 0.001 for *Ipf1/Pdx1*^+/+ ^versus *Ipf1/Pdx1*^-/-^.

**Figure 2:**
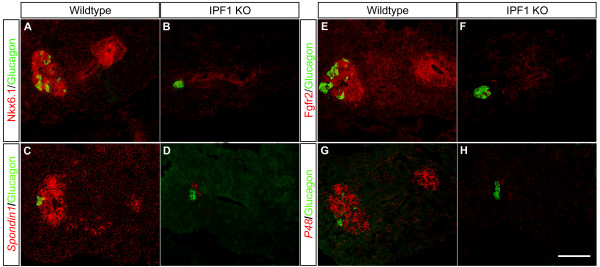
***In situ *and immunohistochemical analyses of differentially expressed genes**. Immunohistochemical analysis of wildtype (A and E) and *Ipf1/Pdx1*^-/- ^mice (B and F) using antibodies against Nkx6.1 (red in A and B), FGFR2b (red in E and F) and glucagon (green in A, B, E and F). *In situ *hybridization of wildtype (C and G) and *Ipf1/Pdx1*^-/- ^mice (D and H) using DIG-labeled *Spon1 *(red pseudocolor in C and D) and *Ptf1a/p48 *(red pseudocolor in G and H) probes counterstained with antibodies against glucagon (green in C, D, G and H). Scale bar 100 μm.

In agreement with the microarray data and previous findings [11], the expression of the *Nkx6.1*, which encode a homeodomain transcription factor that alike *Ipf1/Pdx1 *is expressed in pancreatic progenitors and later in pancreatic β-cells [12-15], was virtually undetectable in e10.5 *Ipf1/Pdx1*^-/- ^dorsal pancreatic buds (Fig. 1). Immunohistochemical analyses confirmed the loss of *Nkx6.1 *expression in e10.5*Ipf1/Pdx1*^-/- ^dorsal pancreatic buds (Fig. 2a, b). In contrast, the expression of the pan-endodermal marker *Foxa2 *was normal in e10.5 *Ipf1/Pdx1*^-/-^dorsal pancreatic buds (Fig. 1) and *Sox9 in situ *hybridization clearly depicted strong expression of *Sox9 *in both the dorsal and ventral pancreatic buds in the mutant embryos (See Additional file 1). These data demonstrate that the difference in gene expression observed in wildtype and *Ipf1/Pdx1*^-/- ^dorsal pancreatic buds do not reflect a general decrease in endodermally expressed genes. The expression of *Pax6*, which encode a transcription factor expressed, and involved, in pancreatic endocrine cell differentiation [16,17] was reduced by ~69% in e10.5 *Ipf1/Pdx1*^-/-^dorsal pancreatic buds (Fig. 1). Nevertheless, glucagon-expressing cells still appear in the *Ipf1/Pdx1*^-/- ^mutant pancreas ([9] and Fig. 2) and *glucagon *expression was largely unaffected in e10.5 *Ipf1/Pdx1*^-/- ^dorsal pancreatic buds (See Additional file 2), providing evidence that the remaining *Pax6 *expression is sufficient to ensure *glucagon *expression. The expression of *ErbB3*, a receptor of the EGF-family that previously has been implicated in pancreatic growth [18,19], was reduced by ~74% in e10.5 *Ipf1/Pdx1*^-/- ^dorsal pancreatic buds (Fig. 1). The expression of two genes encoding extracellular matrix proteins, *Spp1 *and *Spon1*, was reduced by ~91% and ~93%, respectively in e10.5 *Ipf1/Pdx1*^-/- ^dorsal pancreatic buds (Fig. 1). *Spp1*, also known as osteopontin, is known to be expressed in the fetal pancreatic epithelium and although *Spp1 *has been implicated in cell adhesion and migration in several tissues the pancreas develops normally in *Spp1 *deficient mice [20-22]. *Spon1 *encodes the protein F-spondin that is involved in proliferation of vascular SMC, neurite outgrowth and differentiation of neuronal precursor cells [23-29]. The reduced expression of *Spon1 *in e10.5 pancreatic progenitor cells was confirmed by *in situ *hybridization (Fig. 2c, d). The expression of *Mgst1*, which encode an integral membrane protein involved in cellular response to chemical or oxidative stress [30] and *Rtn1*, which encodes a neuroendocrine-specific protein [31], was reduced by 64% and 60%, respectively in e10.5 *Ipf1/Pdx1*^-/- ^dorsal pancreatic buds (Fig. 1).

In contrast, three genes, *Blzf1*, a gene up-regulated by retinoids [32,33], *Schip1*, which is involved in cell cycle regulation [34,35], and *Sfrp1*, which encode a secreted antagonist to Wnt signaling [36], which all were identified as differentially expressed in the microarray analysis could not be validated when analyzed by quantitative real-time RT-PCR and were excluded from further analyses (data not shown). Taken together, these data show that the expression of several genes implicated in growth, differentiation and adhesion, some of which have previously been associated with pancreatic development, is impaired in *Ipf1/Pdx1*^-/- ^pancreatic progenitor cells.

### *Ptf1a/p48 *and *FgfR2IIIb *expression is perturbed in *Ipf1/Pdx1*^-/- ^pancreatic buds

The expression of two genes, *pancreas specific transcription factor 1a/p48 *(*Ptf1a/p48*) and *fibroblast growth factor receptor 2IIIb *(*FgfR2IIIb*), that previously have been implicated to have a role in pancreatic development [37-39] but which were not spotted on the microarray chips were also analyzed by quantitative real-time RT-PCR. *FgfR2IIIb *is expressed in the developing pancreas and in *FgfR2IIIb *null mutant mice pancreatic growth is reduced and the branching and morphogenesis of the pancreatic ductal epithelium is impaired [39]. Quantitative real-time RT-PCR analysis revealed that *FgfR2IIIb *expression was reduced by ~75% in e10.5 *Ipf1/Pdx1*^-/- ^dorsal pancreatic buds compared to that of stage matched *Ipf1/Pdx1*^+/+ ^dorsal pancreatic buds (Fig. 1). Immunohistochemical analyses confirmed the reduced expression of FGFR2IIIb in e10.5 pancreatic progenitor cells of *Ipf1/Pdx1*^-/- ^deficient mice (Fig. 2e, f).

PTF1a/p48 has been implicated in commitment and proliferation of pancreatic progenitor cells and *Ptf1a/p48*^-/- ^null mutant mice fail to develop a pancreas [37,38]. Quantitative real-time RT-PCR analyses showed that *Ptf1a/p48 *expression was reduced by ~91% in *Ipf1/Pdx1*^-/- ^e10.5 dorsal pancreatic buds as compared to wild-type littermates (Fig. 1). *In situ *hybridization analyses confirmed the reduced expression of *Ptf1a/p48 *in dorsal pancreatic buds of *Ipf1/Pdx1*^-/- ^embryos compared to wildtype littermates (Fig. 2g, h). These results suggest that in early pancreatic progenitor cells both *FgfR2IIIb *and *Ptf1a/p48 *are direct or indirect *Ipf1/Pdx1 *downstream target genes.

### *Spon1 *expression in pancreatic progenitor cells overlaps temporally with the phase of proliferation

*Spon1 *encodes the extracellular matrix protein F-spondin, also known as vascular smooth muscle cell growth-promoting factor. F-spondin stimulates the proliferation and growth of vascular SMC during ovarian growth and development [28]. In addition, F-spondin has been reported to promote neurite outgrowth from spinal chord, hippocampal and sensory neurons [23,25,27] as well as the differentiation of neural progenitor cells [29]. *Spon1 *has not previously been described to be expressed in the developing pancreas. Thus, we next determined the temporal and spatial expression of *Spon1 *in the developing pancreas by *in situ *hybridization.

**Figure 3:**
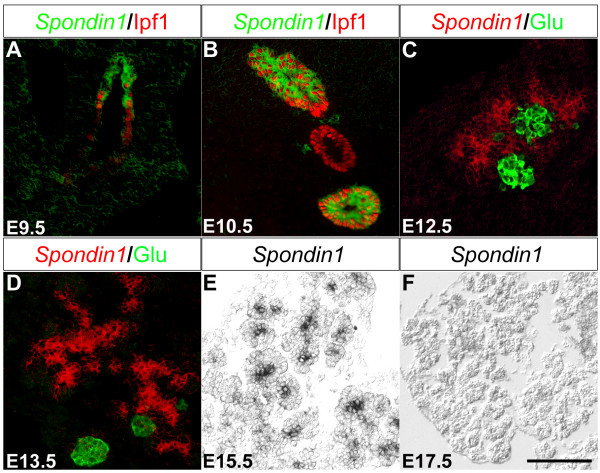
***Spon1 *is expressed in the developing pancreatic epithelium**. A-F: *In situ *hybridization of e9.5 (A), e10.5 (B), e12.5 (C), e13.5 (D), e15.5 (E) and e17.5 (F) using a DIG-labeled *Spon1 *probe (green pseudo-color in A-B, red pseudo-color in C-D and black staining in E) counterstained with antibodies against IPF1/PDX1 (red in A-B) and glucagon (green in C-D). Scale bar 100 μm.

At e9.5 *Spon1 *expression was observed in epithelium of the dorsal and ventral pancreatic bud (Fig. 3a and data not shown). *Spon1 *expression was maintained within the pancreatic epithelium of the developing pancreas until e15.5, although the expression appeared reduced at this stage (Fig. 3b–e). By e17.5 *Spon1 *expression was no longer detectable within the pancreas (Fig. 3f). Throughout pancreatic development *Spon1 *expression was restricted to the developing pancreatic epithelium and the forming acini whereas no expression was observed in differentiated endocrine cells (Fig. 3c, d). In contrast to IPF1/PDX1, *Spon1 *was not expressed in the developing duodenal epithelium (Fig. 3b and data not shown). These data imply that additional transcription factors act to restrict *Spon1 *expression in the developing gastrointestinal tract and also suggest that *Spon1 *may be used as a marker to distinguish between early pancreatic and duodenal cells.

## Discussion

In *Ipf1/Pdx1 *deficient embryos, pancreatic development becomes arrested around e10 [6,7,9] but the molecular mechanism(s) underlying this early developmental arrest has remained largely unknown. To begin to identify genes regulated by *Ipf1/Pdx1 *in early pancreatic progenitor cells that could help to explain the developmental arrest observed in *Ipf1/Pdx1 *^-/- ^embryos we embarked on a gene array profiling and quantitative real-time RT-PCR approach. Given the early developmental arrest and hence limiting amount of tissue, i.e. dorsal buds from e10.5 *Ipf1/Pdx1 *^+/+ ^and *Ipf1/Pdx1 *^-/- ^litter pairs, that could be retrieved for further cDNA synthesis we first performed a pilot study using the 15 K cDNA chip that was hybridized twice with cDNA prepared from dorsal buds of a matched *Ipf1/Pdx1 *^+/+ ^and *Ipf1/Pdx1 *^-/- ^e10.5 litter pair.

This pilot study was followed by two hybridizations of the 15 K cDNA chip with cDNA prepared from another pair of matched *Ipf1/Pdx1 *^+/+ ^and *Ipf1/Pdx1 *^-/- ^e10.5 dorsal buds. The 21 K cDNA chip was originally hybridized with cDNA from 4 pairs of *Ipf1/Pdx1 *^+/+ ^and *Ipf1/Pdx1 *^-/- ^e10.5 dorsal buds in a standard manner. However due to technical problems in the hybridization step one of the arrays had to be excluded from the experiment resulting in a 3 arrays from the 21 K experiment. In summary a total of 4 independent pairs of *Ipf1/Pdx1 *^+/+ ^and *Ipf1/Pdx1 *^-/- ^e10.5 dorsal buds were analyzed on four 15 K arrays and three 21 K arrays.

Hence, there were some limitations in our microarray study. We had a total of four biological replicates per condition: the 15 K experiment used only 2 biological replicates per condition and the 21 K experiment used 3 biological replicates per condition. This limitation was due to challenges in retrieving sufficient tissue material for subsequent cDNA synthesis. We analyzed separately the data from the two arrays, so the results of these analyses are based on a small number of biological replicates. Furthermore, the experimental design was unbalanced in various ways (different types of technical replication in the assays using the 15 K arrays, unbalanced dye assignments, etc.). Thus, the list of differentially expressed genes identified solely by our microarray experiments might be sub-optimal in terms of a bona fide candidate list. However, it provided us with sufficient information for selecting potentially interesting *Ipf1/Pdx1 *regulated genes that we followed-up with quantitative real-time RT-PCR, *in situ *hybridization and immunohistochemistry.

Here we show that the expression of *FgfR2IIIb *is reduced in *Ipf1/Pdx1*^-/- ^e10.5 pancreatic buds, thus linking IPF1/PDX1 to FGF signaling in pancreatic progenitor cells. FGF signaling has been shown to be important both for normal pancreas development and maintenance of pancreatic function in adults [5,40,41]. Mice with a targeted disruption of *Fgf10 *show an early block in pancreas development that is reminiscent of that observed in *Ipf1/Pdx1 *null mutant mice. Alike the *Ipf1/Pdx1*^-/- ^mice the pancreatic program is initiated in *Fgf10*^-/- ^mice, i.e. the evagination of the pancreatic buds occur, but the subsequent proliferation and branching of the pancreatic epithelium is perturbed [42]. Moreover, mice lacking the FGF10 receptor, *FgfR2IIIb*, display a pancreatic growth defect with impaired branching and morphogenesis of the ductal epithelium. [39]. Moreover, forced expression of *Fgf10 *in the developing pancreatic epithelium results in pancreatic hyperplasia, adding further evidence for a role for FGF10-FGFR2IIIb signaling in pancreatic progenitor cell proliferation [40,41].

Taken together, the almost identical pancreatic phenotype observed in mice lacking either *Ipf1/Pdx1*, the receptor *FgfR2IIIb*, or *Fgf10*, and the reduced pancreatic expression of *FgfR2IIIb *in *Ipf1/Pdx1*^-/- ^embryos provide evidence for a role for IPF1/PDX1 in regulating FGF signaling in the developing pancreatic epithelium. Thus, *Ipf1/Pdx1 *appears to regulate pancreatic growth by ensuring high level expression of FGFR2IIIb in pancreatic progenitor cells. Interestingly, *Ipf1/Pdx1 *has also been shown to regulate the expression of another FGF-receptor, FGFR1c, in adult β-cells thereby ensuring normal β-cell function and glucose homeostasis [5].

Epidermal growth factor (EGF) signaling has also been linked to pancreatic growth and development [1,19,43]. Several EGF receptors, including ErbB3, have been shown to be expressed in the developing pancreatic epithelium [19]. In addition, *ErbB3 *has been described to be expressed in the pancreatic mesenchyme [18,19]. Mice lacking a functional *ErbB1/Egfr *gene have mildly perturbed pancreatic growth [44] and mice lacking a functional *ErbB3 *gene present with pancreatic hypoplasia [18,44]. Additional support for a role of the EGF family in pancreas development originates from *in vitro *studies in which EGF was shown to stimulate the growth of e13.5 rat pancreatic epithelium that had been depleted of mesenchyme [45]. Thus, in addition to the reduced expression of *FgfR2IIIb *in progenitor cells of *Ipf1/Pdx1 *deficient mice the reduced expression of *ErbB3 *is likely to contribute to the perturbed pancreatic development that these mice present with.

*Ptf1a/p48 *expression is severely reduced in *Ipf1/Pdx1 *null mutant mice, providing evidence that *Ptf1a/p48*, directly or indirectly, is a downstream target gene of *Ipf1/Pdx1*. In support of a role for *Ipf1/Pdx1 *in regulating *Ptf1a/p48 *expression, inactivation of the *Ipf1/Pdx1 *gene in the pancreatic epithelium at progressively later stages also results in reduced expression of *Ptf1a/p48 *[46]. In contrast, Kawaguchi et al. have shown that transgenic expression of *Ipf1/Pdx1 *under the control of the *Ptf1a/p48 *promoter in an *Ipf1/Pdx1*^-/- ^null background partially rescued the pancreatic developmental arrest [37], suggesting that *Ptf1a/p48 *expression is independent of *Ipf1/Pdx1*. However, the *Ptf1a-Ipf1/Pdx1 *transgenic rescue approach leaves open the possibility that the partially rescued phenotype is the result of leaky transgene expression due to positional integration effects and/or transgene copy number. Moreover, since the *hsp*68 minimal promoter was coupled to the *Ptf1a/p48 *promoter in the transgenic rescue approach, the transgene expression may differ from that of the endogenous *Ptf1a/p48 *promoter.

Alike *Ipf1/Pdx1*, *Nkx6.1 *is expressed in pancreatic progenitors from embryonic day 9.5 and thereafter the expression of *Nkx6.1 *also becomes restricted to mature β-cells. *Nkx6.1 *has previously been identified as a downstream target gene of IPF1/PDX1; β-cell specific inactivation of the *Ipf1/Pdx1 *gene resulted in a loss of *Nkx6.1 *expression in β-cells [4,11] and in agreement with previous findings [4,11] we show that *Nkx6.1 *expression is abolished also in pancreatic progenitor cells of *Ipf1/Pdx1 *null mutant mice. These data provide strong evidence that *Ipf1/Pdx1 *act upstream of *Nkx6.1 *in both pancreatic progenitor cells and adult β-cells.

F-spondin, encoded by *Spon1*, is a 90 kDa extracellular matrix protein that has been reported to play an important role in proliferation and growth of vascular SMC during ovarian growth and development [28]. F-spondin has also been showed to promote neurite outgrowth from spinal chord, hippocampal, and sensory neurons [23,25,27]. Other reports describe a role for F-spondin in the development of the nervous system where it is predominately expressed during early stages of development but down-regulated during neural differentiation [23]. F-spondin has also been suggested to promote differentiation of neural precursor cells to nerve cell-like cells [29]. Interestingly, a F-spondin related protein, R-spondin, has been shown to function as a growth factor; injection of R-spondin in mice resulted in an increased proliferation of intestinal crypt epithelial cells in both the small intestine and colon [47]. Similar to the transient expression of *Spon1 *in the developing nervous system, *Spon1 *is transiently expressed, from e9.5 to e15.5 in progenitor cells of the developing pancreas. Thus, *Spon1 *expression in the developing pancreas overlaps with the phase of pancreatic progenitor cell proliferation. Whether F-spondin is involved in regulation of proliferation and/or differentiation of pancreatic progenitor cells will have to be determined by functional analyses *in vitro *and/or *in vivo*.

## Conclusion

Taken together, our data show that *Ipf1/Pdx1*, directly or indirectly, regulates the expression of genes, such as *Ptf1a/p48, FGFR2IIIb*, and *ErbB3*, implicated in pancreatic growth and differentiation, providing evidence that the perturbed expressions of these factors provide a mechanistic explanation for the pancreatic developmental arrest that *Ipf1/Pdx1*^-/- ^mice present with. In addition we show that *Spon1*, which encodes an extracellular matrix protein, is expressed in the developing pancreas and that *Spon1 *expression is down-regulated in *Ipf1/Pdx1*^-/- ^embryos.

## Methods

### Mice

The animal studies were approved by the Institutional Animal Care and Use Committee of Umeå University and conducted in accordance with the Guidelines for the care and use of Laboratory Animals. The generation of *Ipf1/Pdx1*^-/- ^mice has been previously described [6]. *Ipf1/Pdx1 *wildtype and null mutant embryos were obtained from our local breeding stock.

### Isolation of embryos and tissue

*Ipf1/Pdx1*^+/- ^mice were mated and embryonic day 0.5 (e0.5) was determined by the presence of morning vaginal plug. Pregnant females were sacrificed at e10.5 and embryos were transferred to ice-cold Lebovitz L-15 medium (Gibco). Dorsal pancreatic buds were dissected out in L-15 medium and snap frozen in liquid nitrogen for later RNA isolation. Embryos sacrificed for immunohistochemistry and *in situ *hybridization were fixated in 4% paraformaldehyde (SIGMA) in 0.1 M PBS for 2 hours and overnight respectively at +4°C followed by dehydration in 20% sucrose overnight at 4°C and mounted in Tissue-Tek (Sakura) and stored at -80°C.

### Genotyping

Genomic DNA was extracted from embryonic tails by proteinase K (Boehringer) digestion followed by 2-propanol precipitation. Genotyping was performed by PCR using two internal *Ipf1/Pdx1 *primers; 5'-AGAAGCTGGCCACTAGCCTCT-3' and 5'-CTGTGGGCAACAAGGGAGTT-3', and two internal *Neo *primers; 5'-AGAAGCTATTCGGCTATGA-3' and 5'-TTTCCACCATGATATTCG-3'. The PCR reactions result in a 150 bp and a 548 bp product respectively if target sequence is present.

### Microarray, hybridization and scanning

The cDNA microarrays used in this study were of two types. The first contain approximately 15,000 clones printed in duplicate, in two identical fields, yielding 30,000 probes. The set of probes represents approximately 12,000 unique genes obtained through large-scale, in-house EST sequencing of three cDNA libraries. The first library originates from a neural tissue stem cell compartment (lateral ventricular wall), the second from neurospheres (neural stem cells cultured in vitro) and the third from a hematopoietic stem cell line expressing the Lhx2 gene [10]. The second cDNA array used in this study contains 20,600 clones derived from two different clone sets: a 15,000 mouse cDNA set from National Institute of Aging (NIH) and a 5,400 cDNA clone set obtained from Research Genetics. Microarray slides were hybridized at 42°C in a water bath for 16–20 hours (15 K array) or at 37°C in a Genetac hybridization station (Genomic Solutions) for 15 hours (21 K array). The 15 K arrays were scanned using the G2565BA DNA microarray scanner (Agilent Technologies) and images analyzed in the GenePix5.0 software (Axon Instruments, CA, USA). The 21 K arrays were scanned using the Scanarray 4000 XL microarray scanner (Perkin Elmer) at three settings (laser power/PMT): 80/80, 90/90 and 100/100 (in that order), where the numbers were percentages of maximum values. Images were analyzed using the ScanarrayExpress software (Perkin Elmer).

### RNA isolation, amplification and labeling

Total RNA was isolated from individual pancreatic buds using Nucleospin RNA II kit (Clontech) according to manufacturer's guidelines. cDNA was generated and amplified using SuperSMART™ PCR cDNA synthesis kit (Clontech). 2 μg of each cDNA preparation was labeled with the fluorescent dyes Cy3 or Cy5 using BD Atlas™ SMART™ Fluorescent Probe Amplification kit (BD Biosciences Clontech) according to manufacturer's recommendations. Samples hybridized to the 15 K cDNA microarrays were labeled and analyzed as follows: In the first set, the labeled samples (one wildtype bud cDNA labeled with Cy3, one *Ipf1/Pdx1 *null mutant pancreatic bud cDNA labeled with Cy5) were split in two, and analyzed as duplicates on two separate microarrays. In the second set, the samples (one wildtype bud cDNA and one *Ipf1/Pdx1 *null mutant pancreatic bud cDNA) were split before labeling, and analyzed as a dye-swapped replicate on two separate microarrays.

Samples hybridized to the 21 K cDNA microarrays were labeled and analyzed as follows: two wildtype and one *Ipf1/Pdx1 *null mutant pancreatic bud were individually labeled with Cy3 and two *Ipf1/Pdx1 *null mutants and one wildtype pancreatic bud were individually labeled with Cy5. Samples were pooled pair wise and hybridized to three 21 K cDNA microarrays.

One wt cDNA sample and one *Ipf1/Pdx1 *null mutant cDNA sample hybridized to the 21 K array were identical to second set of samples hybridized to the 15 K array. Due to the limited amount of material, the rest of the samples were hybridized to one array each (15 K or 21 K array).

In total, cDNA generated from pancreatic buds from 4 individual *Ipf1/Pdx1 *null mutant and 4 wildtype littermates respectively were individually labeled, pooled pair wise and hybridized to four 15 K cDNA microarrays and to three 21 K cDNA microarrays.

### Microarray data processing and statistical analysis

The 15 K and 21 K experiments were analyzed separately as described below. Given some selection criteria, discussed below, 111 genes were considered differentially expressed. 73 of these genes were down-regulated (Table 1) and 38 up-regulated (Table 2) in *Ipf1/Pdx1 *null mutants. Additional information and all array data are reported to ArrayExpress [48] with the following accession numbers. [ArrayExpress: E-TABM-280] and [ArrayExpress: E-MEXP-1047].

The 21 K microarray analyses were carried out using ScanArrayExpress 2.1 (PerkinElmer) and the in-house S-Plus library UmeaSAMED 1.39. The data analysis included: combined image analysis extracting median intensity values, complete filtration, local background correction, RLS 100, removed C-spots, MA-print-tip normalization with span = 0.5, censoring with minimum value 8, and the B-test, see [49] for detailed descriptions of the procedures. Genes with top-ranked B-statistics (top 100) and at least two-fold regulated (i.e. the absolute value of the average log-ratio 1), were selected for further studies.

The 15 K microarray analyses were carried out using R (1.7), Bioconductor, LIMMA (1.3), and the aroma package [50-52]. Several filtering steps, normalization and statistical analyses were done, as detailed in ArrayExpress. The foreground intensities without background subtraction were extracted using the median intensity values. A feature was filtered if it was flagged as "Not Found" in GenePix, if both channels were saturated, the percentage of foreground pixels above the local background plus two of its standard deviations were below 70 for both channels, or the signal-to-noise ratio for both channels were below 3. Data was normalized using MA-print-tip normalization [53]. The intensities for duplicated features were averaged after normalization. The empirical Bayes moderated t-test implemented in the LIMMA package (*i.e*. the B-test) was used [54] to identify differentially expressed genes [51]. The four 15 K assays were analyzed in two separate sub-experiments, each including the two assays derived from the same biological replicates. Genes that were at least two-fold up-regulated (or down-regulated) in both sub-experiments and had B-values larger than 10 in sub-experiment 1 and 2 in sub-experiment 2 were selected for further studies.

### Quantitative real- time RT-PCR

Gene expression levels were measured using the ABI PRISM 7000 Sequence Detection System (Applied Biosystems) according to the manufactures recommendations. Expression of the β-2-microglobulin (*b2M *and *TBP*) was used to normalize expression levels. Primer sequences were the following: *B2M*: 5'-GCTATCCAGAAAACCCCTCAAA-3', 5'-CTGTGTTACGTAGCAGTTCAGTATGTTC-3'; *Blzf1*: 5'-ACAGCCCAGCTTTCTGAACAG-3', 5'-ACTCATCTGCCATTACCCTGCTT'3'; *ErbB3*: 5'-CTCTCCCCACCAGGGTTAGAG-3', 5'-TGACGAAAGGGTCCCTTCC-3'; *Fgfr2IIIb*: 5'-CAGTAAATACGGGCCTGATGG-3', 5'-GAACAGAGCCAGCACTTCTGC-3'; *Foxa2*: 5'-GAGCCATCCGACTGGAGCA-3', 5'-GGAATGAGCCCGTCGCTAG-3'; *Glu*: 5'-CCGCCGTGCCCAAGA-3', 5'-CATCATGACGTTTGGCAATGTT-3'; *Ipf1*: 5'-TAGGACTCTTTCCTGGGACCAA-3', 5'-AATAAAAAGGGTACAAACTTGAGCGT-3'; *Mgst1*: 5'-CGCATTCCAGAGGATAACCAA-3', 5'-CAAAGCCAGCGCAGTCTTCT-3'; *Nkx6.1*: 5'-TCAGGTCAAGGTCTGGTTCCA-3', 5'GGTCTCCGAGTCCTGCTT-3'; *P48*: 5'-TCATCTGCCATCGAGGCAC-3', 5'-ACCATAATCCGGGTCACTGG-3'; *Pax6*: 5'-GCAAACAACCTGCCTATGCAA-3', 5'-GGCAGCATGCACGAGTATGA-3'; *Reticulon*: 5'-CCTGGTCCAGGACCTGGTG-3', 5'-AGCCACATGAGGACTGCAAAT-3'; *Schip1: *5'-AAAAAGTCTCCCGTCGCTGA-3', 5'-TTCGCTTATGTGAGGCATGTGT-3'; *Sfrp1*: 5'-CCTCCATGCGACAACGAGTT-3', 5'-TTGATTTTCATCCTCAGTGCAAAC-3'; *Spondin1*: 5'-GAAGCAGCTACCGAGTGACACTT-3', 5'-CTTTGAGGGCAATTAACGTGAAA-3'; *Spp1*: 5'-TCAAAGTCTAGGAGTTTCCAGGTTTC-3', 5'-TGTGGCATCAGGATACTGTTCATC-3'; *TBP*: 5'-GAATTGTACCGCAGCTTCAAAA-3', 5'-AGTGCAATGGTCTTTAGGTCAAGTT-3'.

### Immunohistochemistry

Immunohistochemistry was performed as described [9]. Primary antibodies used were: rabbit anti-IPF1/PDX1 [3], rabbit Nkx6.1 [40], rabbit anti-FgfR2 (Research Diagnostics, Inc) and glucagon anti-guinea pig (Electra-Box Diagnostika Ab). Secondary antibodies used were: Cy3 anti-rabbit (Jacksson) and Alexa 488 anti-guinea pig (Molecular Probes).

### *In situ *hybridization

*In situ *hybridization was performed as described [9]. Probes used were: *Ptf1a/p48 *(provided by O. Hagenbüchle), *Sox9 *(covering region 1365–2305 bp of image clone 5320371) and *Spon1 *(covering region 1237–4035 bp of image clone 4221758).

## Authors' contributions

PS and IB performed the experiments and drafted the manuscript. CW, JL, and PR, helped with the microarray and bioinformatics. HE helped design and supervise the work and write the paper. All authors read and approved the final manuscript.

## Supplementary Material

Additional File 1 ***In situ *hybridization of wildtype and *Ipf1/Pdx1*^-/- ^mice respectively**. *In situ *hybridization of wildtype (A) and *Ipf1/Pdx1*^-/- ^(B) e10.5 mice using a DIG-labeled *Sox9 *probe (red pseudo-color in A and B) counterstained with antibodies against glucagon (green in A and B). Scale bar 100 μm. Click here for file

Additional File 2 **Quantitative real-time RT-PCR of glucagon**. Expression analysis of glucagon using cDNA from *Ipf1/Pdx1*^+/+ ^(n = 6) and *Ipf1/Pdx1*^-/- ^(n = 6) dorsal e10.5 pancreatic buds. Data represent mean values ± SEM. Click here for file
