# Supplementary material for: Gene array identification of Ipf1/Pdx1-/- regulated genes in pancreatic progenitor cells
Source: BMC Dev Biol. 2007 Nov 23;7:129. doi: 10.1186/1471-213X-7-129 (PMC2212654; doi:10.1186/1471-213X-7-129)
Supplement: Additional File 1 — In situ hybridization of wildtype and Ipf1/Pdx1-/- mice respectively. In situ hybridization of wildtype (A) and Ipf1/Pdx1-/- (B) e10.5 mice using a DIG-labeled Sox9 probe (red pseudo-color in A and B) counterstained with antibodies against glucagon (green in A and B). Scale bar 100 μm. [file 1471-213X-7-129-S1.doc]

**Additional file 1**

**
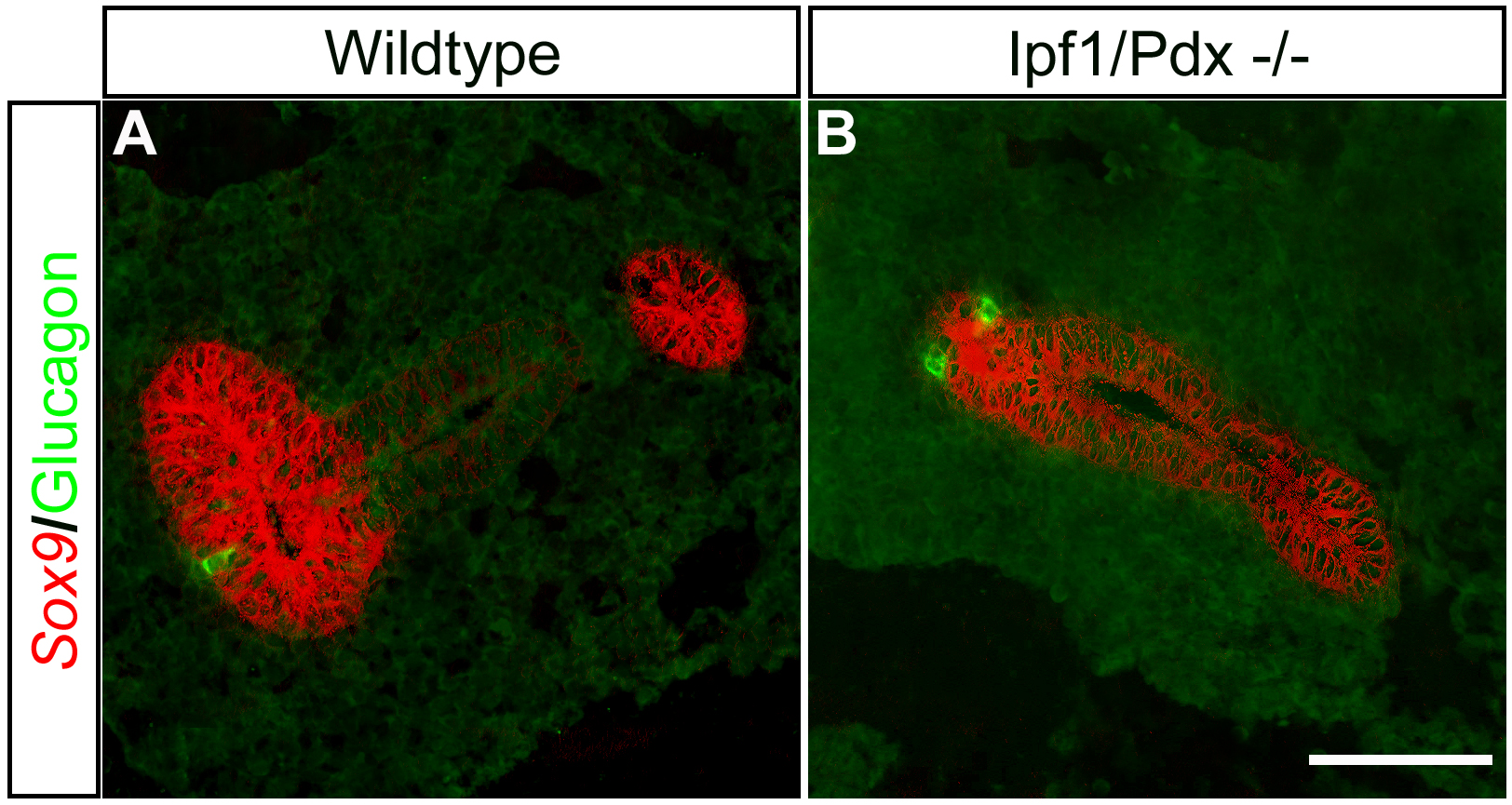
**

***In situ* hybridization of wildtype and *Ipf1/Pdx1*^-/-^ mice respectively.** *In situ* hybridization of wildtype (A) and *Ipf1/Pdx1*^-/-^ (B) e10.5 mice using a DIG-labeled *Sox9* probe (red pseudo-color in A and B) counterstained with antibodies against glucagon (green in A and B). Scale bar 100μm.
