# Supplementary material for: Gene array identification of Ipf1/Pdx1-/- regulated genes in pancreatic progenitor cells
Source: BMC Dev Biol. 2007 Nov 23;7:129. doi: 10.1186/1471-213X-7-129 (PMC2212654; doi:10.1186/1471-213X-7-129)
Supplement: Additional File 2 — Quantitative real-time RT-PCR of glucagon. Expression analysis of glucagon using cDNA from Ipf1/Pdx1+/+ (n = 6) and Ipf1/Pdx1-/- (n = 6) dorsal e10.5 pancreatic buds. Data represent mean values ± SEM. [file 1471-213X-7-129-S2.doc]

**Additional file 2**

**Quantitative real-time RT-PCR of glucagon.** Expression analysis of glucagon using cDNA from *Ipf1/Pdx1*^+/+^ (n=6) and *Ipf1/Pdx1*^-/-^ (n=6) dorsal e10.5 pancreatic buds. Data represent mean values ± SEM.
